# Therapy-Associated Myeloid Dysplasia in a Long-Surviving Patient with Pancreatic Cancer

**DOI:** 10.7759/cureus.687

**Published:** 2016-07-13

**Authors:** Jillian Suzukida, Kristin Kaley, Azra Raza, Muhammad W Saif

**Affiliations:** 1 Internal Medicine, Tufts Medical Center; 2 Yale Cancer Center, Yale School of Medicine; 3 Myelodysplastic Syndrome Center, Columbia University; 4 Hematology/Oncology, Tufts Medical Center

**Keywords:** mds, leukemia, pancreatic cancer, oxaliplatin, radiation

## Abstract

Pancreatic cancer remains a diagnosis of poor prognosis with a median survival time of four-six months in patients with advanced stage of the disease. Although, with the development of novel chemotherapy agents some patients are able to live a little longer if they respond to therapy. However, long-term complications of chemotherapy or radiotherapy are not known due to the short survival period of patients with pancreatic cancer. We present a case of a 55-year-old-woman who developed therapy-related myelodysplastic syndrome (t-MDS) during a survival of approximately eight years during which she received multiple chemotherapies and radiation therapy. She presented with progressive fatigue and pancytopenia, which led to further work-up and led to the diagnosis of t-MDS. The latency period to developing hematologic abnormalities as well as the presence of the chromosome 5 and 7 abnormalities in this patient are likely consistent with t-MDS and possibly related to the use of chemotherapeutic agents such as oxaliplatin or irinotecan and radiation therapy.

## Introduction

Pancreatic cancer is diagnosed in approximately 44,000 people each year in the United States, and is the fourth leading cause of cancer-related death in both males and females [1]. Surgery is the only potential cure, but less than 20% of patients present with localized tumors. The overall five-year survival rate is less than five percent. Patients diagnosed with metastatic pancreatic cancer have a median survival of four-six months. First line treatment has traditionally been single agent gemcitabine since a study in 1997 showed improved measures of quality of life (pain, weight, and Karnofsky performance status) when compared with 5-fluorouracil [2]. The use of gemcitabine also resulted in slight improvement in median overall survival (5.65 vs. 4.41) and one-year survival rates (18% vs. 2%). Gemcitabine plus erlotinib has also been approved as a treatment option for metastatic cancer, based on a study that showed minor improvements in median overall survival (6.24 vs. 5.91 months) as well as one-year survival rate (23% vs. 17%) [1]. Since this treatment option comes at a cost of increased toxicities, including rash and interstitial lung disease, this regimen failed to become an acceptable one among treating oncologists. In 2011, a study comparing FOLFIRINOX (5-fluorouracil, leucovorin, irinotecan, and oxaliplatin) to gemcitabine showed an increase of overall survival (11.1 vs. 6.8 months) in patients with metastatic pancreatic cancer with Eastern Cooperative Oncology Group (ECOG) performance scores 0-1 [3]. Most recently, the Metastatic Pancreatic Adenocarcinoma Clinical Trial (MPACT) study showed the survival benefit of combining gemcitabine with nab-paclitaxel over gemcitabine alone [4]. In addition, multiple Phase II and Phase III studies have at least indicated the role of a few other drugs in selected cases but failed to show any superiority over gemcitabine in randomized studies.

We report here the case of a long-term survivor of pancreatic cancer who developed therapy-related myelodysplastic syndrome (t-MDS) seven years after initial diagnosis and a review of the literature. Informed consent was obtained from the patient for this study.

## Case presentation

A 55-year-old Caucasian woman presented in 2005 with jaundice, abdominal pain, light-colored stools, dark urine, and pruritus. At the time of diagnosis, her past medical history was only significant for splenic artery aneurysm, congenital atrophic right kidney, and remote history of a right wrist ganglion cyst removal. She had no known allergies, had never used tobacco, and she reported rare alcohol use. Her family history was significant for a brother who passed at age 12 from possible renal failure and another brother with congenital deafness. Her mother and father passed away at ages 85 and 95 respectively. Her mother died from cancer but the patient did not remember the primary site. She had three children as well as an identical twin sibling. 

Pancreatic adenocarcinoma was diagnosed as stage pT3N1 following an endoscopic retrograde cholangiopancreatography (ERCP) and endoscopic ultrasound (EUS). A Whipple procedure was performed and the pathology showed moderate to poorly differentiated ductal adenocarcinoma, involving the pancreas, stomach, duodenum, common bile duct, and peripancreatic soft tissue, positive for vascular and perineural invasion, and involvement of three out of seven lymph nodes. She was initially treated with six cycles of adjuvant gemcitabine starting in 2005. In 2006, a positron emission tomography and computed tomography (PET-CT) scan showed evidence of recurrence as a soft tissue mass and lymph nodes in the surgical bed, accompanied with a rise in CA19-9 to 440 IU. She was treated with 12 cycles of FOLFOX (folinic acid, 5-fluorouracil, oxaliplatin) plus bevacizumab (bevacizumab was discontinued on the eighth cycle due to upper gastrointestinal bleeding) and the tumor markers normalized.

During 2007, the CA19-9 rose again and recurrence was seen in the surgical bed on PET-CT. Seventeen cycles of gemcitabine plus oxaliplatin (GEMOX) and bevacizumab were administered with partial response. In August 2008, a CT scan showed new pleural-based nodules suspicious for metastases, which were confirmed histologically. Then she was started on irinotecan after UGT1A1 gene polymorphism was negative for UGT1A1*28 polymorphism. She received a total of 16 cycles of irinotecan from September 2008 to April 2009 with normalization of CA19-9. She was then followed closely. However, irinotecan was restarted in December 2009 after the CA19-9 level began to rise again. The CA19-9 level continued to increase despite treatment with irinotecan and a repeat PET-CT showed recurrence at the surgical site once again. Fractionated external beam radiation at a dose of 4860 cGy was started in April 2010, followed by capecitabine plus mitomycin (MIXE) for six cycles starting in June 2010. The patient was given chemotherapy holiday and followed closely with scans and tumor markers.

In the spring of 2012, the patient began complaining of progressive fatigue with dyspnea on exertion. Blood work showed mild cytopenias: white blood cell (WBC) count 1.7 K/mcL, hemoglobin (Hgb) 8.1 g/dL, hematocrit (HCT) 25%, and platelet count of 79,000/mcL. Iron supplements were initially started, followed by Vitamin D without improvement. Symptoms of fatigue progressed, accompanied by lightheadedness and exertional dyspnea. Repeat blood work showed persistent cytopenias in July 2012: WBC count 1.0 K/mcL (absolute neutrophil count: 440), Hgb 6.4 g/dL, HCT 20.1%, and platelet count of 70,000/mcL. She was admitted to the hospital and additional testing including bone marrow aspiration and biopsy were performed. Reticulocyte count was 2.3%, iron was 95, TIBC 330, ferritin 132, vitamin B12 668, and RBC folate 1056. Liver function tests were within normal limits and LDH was 161 IU. Reticulated platelet count was 12%, corresponding to a platelet count of 60,000/mcL. She was transfused with two units of packed red blood cells (PRBCs) with improvement in symptoms. Bone marrow biopsy normocellular marrow was 40% with myelodysplasia and CD34 and CD117 positive blasts ranging 6–9%, interpreted as on the transition between refractory anemia with excess blasts-1 (RAEB-1) and RAEB-2. The marrow flow cytometry showed an abnormal CD10/CD13/CD16/CD11b myeloid maturation pattern. A fluorescence in situ hybridization (FISH) test showed deletions of 5q and 7q in 75%–81% of bone marrow cells, and cytogenetics showed an abnormal clone with deletions of 5q, 7q, and 12p, losses of one copy of chromosome of chromosomes 17, 18, 21, and 22, and two to three marker chromosomes. She was evaluated at two major hematologic centers, whose experts both agreed with the diagnosis of therapy-related myelodysplastic syndrome (t-MDS). 

Given the patient’s high risk features, it was decided that treatment for MDS should be performed, and she was enrolled in a clinical trial of decitabine and all-trans retinoic acid (ATRA) in August 2012. A repeat bone marrow biopsy following Cycle 2 showed one percent blasts, decreased from eight percent at diagnosis, with an excellent platelet response.

She was last seen in the oncology clinic in June 2013 when imaging and laboratory data showed stable pancreatic cancer lesions and stable CA19-9. She continued to be variably pancytopenic, however, the platelet count was near normal: 140,000/cmL. At that time, she was closely followed by her hematologists. She told us that she has been taken off the ATRA/decitabine trial due to persistent transaminitis and later only continued on decitabine after her transaminitis had resolved. We scheduled a follow-up with us in three months but unfortunately she died in two months from her last visit. The cause of her death was related to complications arising from t-MDS.

## Discussion

The overall five-year survival for all stages of pancreatic cancer is around seven percent and for metastatic stage it is only two percent [1]. These rates make our patient’s survival up to eight years past diagnosis despite multiple recurrences unique. But the case also presented new challenges in the form of possible treatment-related complications, such as t-MDS in this patient. Such complications have not been seen previously because the majority of the patients either live for a short span or their performance status does not allow continued chemotherapy or multiple lines of chemotherapy agents. This situation adds further challenges because pancreatic cancer is relatively a chemo-resistant tumor and if chemotherapy is helping a patient, we are very reluctant to stop treatment. Though this patient received many treatment breaks in-between due to outstanding radiological and tumor marker responses, overall, she received many years of chemotherapy including platinum drugs and topoisomerase poisons.

Therapy-related myeloid neoplasms (t-MN), including acute myeloid leukemias (AML) and myelodysplastic syndromes (t-MDS), were first fully recognized as a distinct entity by the World Health Organization (WHO) in 2001 [5]. They are reported to develop following treatment with chemotherapy, radiation therapy, immunosuppressive agents, or after exposure to environmental carcinogens such as benzene. Therapy-related MDS accounts for 10%–20% of cases of MDS or acute myelogenous leukemia (AML).

For our patient, the latency period to developing t-MDS was about seven years. The chronological association of prior chemotherapy regimens administered in this patient as well as the cytogenetic analysis showing chromosomes 5 and 7 abnormalities support the diagnosis of treatment-related MDS. Therapy-related MDS is classically associated with exposure to alkylating agents or radiation, with an average latency period of five to seven years, and is also associated with chromosome 5 and 7 abnormalities [6]. Following the use of topoisomerase II inhibitors such as etoposide, doxorubicin, epirubicin there is a shorter latency period of one to three years after chemotherapy that leads to leukemic transformation [7]. Cytogenetic abnormalities associated with topoisomerase inhibitors are 11q26, t(9;11), or 21q22 [7]. Recent literature also shows case reports of patients with MDS/AML possibly caused by damage to DNA or DNA inter-strand crosslink formation following exposure to oxaliplatin and irinotecan [8-9]. Oxaliplatin is a third generation platinum analogue, and has been linked to t-MN, unlike other platinum agents such as cisplatin and carboplatin [9]. It is possible that this patient's t-MDS may also be related to radiation therapy received in addition to chemotherapy.

​There is currently no standard treatment for t-MDS, as few clinical trials include these patients. Retrospective studies have shown that t-MDS/AML does respond to standard induction therapy for AML as well as hypomethylating agents for MDS. Moreover, similar to de novo disease, stem cell transplant is the only curative option for these patients who developed t-MDS [10]. As a form of relatively low-intensity chemotherapy, the DNA methyltransferase inhibitor (DMTI) hypomethylating agents such as 5-azacytidine (AzaC) and decitabine (5-aza-2′-deoxycytidine) have been shown in randomized Phase III trials to decrease the risk of leukemic transformation and, in a portion of the patients, improve survival. Because data has predominantly indicated altered natural history and decreased evolution to AML in patients who experience response to treatment, the major candidates for these drugs are patients with International Prognostic Scoring System (IPSS) intermediate - 2 risk (INT-2) or high-risk MDS, such as:

1- Those who are not candidates for high-intensity therapy;

2- Those who are potential candidates for allogeneic hematopoietic stem cell transplantation (HSCT) but for whom delay in receipt of that procedure is anticipated (e.g., because of time needed to further reduce the blast count, improve the patient’s performance status, or identify a donor). In these circumstances, the drugs may be used as bridging therapy for that procedure; and

3- Those who experience relapse after allogeneic HSCT.

However, compared to patients presenting with de-novo MDS/AML, t-MN patients often enter therapy with reduced reserves resulting from chemotherapy or radiation-induced parenchymal or vascular toxicity, surgical or functional loss of organ function from the primary malignancy, or reduced stem cell reserves or marrow stroma damage, often rendering lower intensity therapeutic options necessary.

Though it is not easy to accept with only anecdotal reports the association of MDS with previous treatment in patients who received oxaliplatin or irinotecan, the fact is that most patients with pancreatic cancer live shorter and if they have an undiagnosed genetic predisposition to both diseases it makes treatment even more challenging. In our experience with colorectal cancer patients, thousands of them have been treated so far with FOLFOX and/or FOLFIRI and no such red flag has been raised to date in the literature except a few published case reports. However, it is also possible that the degree of suspicion is low as physicans may attribute myelosuppression to chemotherapy directly instead of considering a leukemic or pre-leukemic condition caused by the chemotherapy agents in these patients. Moreover, it is also a fact that our patient showed deletions of 5q and 7q in 75%–81% of bone marrow cells and an abnormal clone with deletions of 5q, 7q, and 12p, losses of one copy of chromosome of chromosomes 17, 18, 21, and 22, and two to three marker chromosomes—all consistent with t-MDS.

Therapy-related MDS carries a worse prognosis in this population despite treatment. In addition, an allogeneic bone marrow transplant that is the only curative approach to this disease will not be considered an optimal option for patients with Stage IV pancreatic cancer.

## Conclusions

In summary, for this patient, the five-to-seven-years latency period to developing t-MDS as well as the presence of chromosome 5 and 7 abnormalities are likely consistent with t-MDS related to the use of chemotherapy agents, such as oxaliplatin. This patient's t-MDS may also be related to radiation therapy received. As in the treatment of myeloid neoplasms, stem cell transplant is the only curative option for t-MDS, with inferior results seen in those aged > 35 years, with poor-risk cytogenetics, uncontrolled disease, or a non-sibling related or mismatched unrelated donor. Awareness of this potential hematologic complication of systemic chemotherapies in patients surviving longer is of importance, and in addition, it underlines the significance of developing novel agents to treat cancer with least risk to develop MDS.
